# Comparison of the Pathway to Hospice Enrollment Between Medicare Advantage and Traditional Medicare

**DOI:** 10.1001/jamahealthforum.2022.5457

**Published:** 2023-02-17

**Authors:** Claire K. Ankuda, Emmanuelle Belanger, Jennifer Bunker, Pedro Gozalo, Laura Keohane, David Meyers, Amal Trivedi, Joan M. Teno

**Affiliations:** 1 Department of Geriatrics and Palliative Medicine, Icahn School of Medicine at Mount Sinai, New York, New York; 2 Brown School of Public Health, Brown University, Providence, Rhode Island; 3 Department of Health Policy, Vanderbilt University Medical Center, Nashville, Tennessee

## Abstract

**Importance:**

Older adults in Medicare Advantage (MA) enroll in hospice at higher rates than those in traditional Medicare (TM), but it is unclear whether the pathway of care prior to hospice use differs between MA and TM.

**Objective:**

To examine the site of care prior to hospice enrollment for MA beneficiaries compared with those in TM.

**Design, Setting, and Participants:**

This population-based, retrospective cross-sectional study used Medicare claims data for decedents in calendar years 2011, 2013, 2016, and 2018 who enrolled in hospice in the last 90 days of life. Data were analyzed from February 11, 2022, to October 24, 2022.

**Exposures:**

Enrollment in MA or TM in the last month of life.

**Main Outcomes and Measures:**

The main outcome was the site of care prior to hospice enrollment, defined as hospital, nursing home, and home with or without home health, dichotomized as community vs hospital in a logistic regression model. Covariates included decedent demographics, hospice primary diagnosis, and county-level MA penetration. Differences in hospice length of stay between MA beneficiaries and TM beneficiaries were assessed using linear and logistic regression models.

**Results:**

In this study of 3 164 959 decedents, mean (SD) age was 83.1 (8.6) years, 55.8% were female, and 28.8% were enrolled in MA. Decedents in MA were more likely to enroll in hospice from a community setting than were those in TM, although the gap narrowed over time from an unadjusted 11.1% higher rate of community enrollment in MA vs TM in 2011 (50.1% vs 39.0%) to 8.1% in 2018 (46.4% vs 38.3%). In the primary adjusted analysis over the entire study period, MA enrollment was associated with an 8.09–percentage point (95% CI, 7.96-8.21 percentage points) higher rate of hospice enrollment from the community vs all other sites. This association remained in multiple sensitivity analyses to account for potential differences in the populations enrolled in MA vs TM. The mean overall hospice length of stay was 0.29 days (95% CI, 0.24-0.34 days) longer for MA decedents compared with TM decedents.

**Conclusions and Relevance:**

Compared with TM beneficiaries, those in MA were more likely to enroll in hospice from community settings vs following inpatient stays. However, hospice length of stay was not substantially different between MA and TM. Further research is needed to understand how MA plans influence hospice use and the direct association with quality of end-of-life care as reported by older adults and their families.

## Key Points

**Question:**

Does the site of care prior to hospice enrollment differ between Medicare Advantage (MA) and traditional Medicare (TM)?

**Findings:**

In this cross-sectional study of 3 164 959 decedents, MA enrolled a higher percentage of decedents in hospice compared with TM. Decedents enrolling in hospice in MA were an adjusted 8.09 percentage points more likely to be in the community vs hospital or nursing home settings prior to hospice enrollment, although the difference between TM and MA in mean hospice length of stay was only 0.29 days longer for MA enrollees.

**Meaning:**

In this study, decedents in MA were more likely to enroll in hospice from a community setting but had similar lengths of stay in hospice compared with those in TM.

## Teaser

This cross-sectional study uses Medicare claims data from decedents to compare the site of care before hospice enrollment between Medicare Advantage beneficiaries and traditional Medicare beneficiaries.

## Introduction

In the past 2 decades, hospice enrollment among Medicare beneficiaries has increased markedly. In 2000, 22.9% of decedents enrolled in Medicare received hospice care at the end of life compared with 51.6% in 2019.^1,2^ Hospice care is associated with higher quality of care at the end of life^3,4^ and lower health care costs (including out-of-pocket costs for families).^3,5^ However, many individuals only enter hospice care very late in life; in 2018, 27.9% of hospice beneficiaries were enrolled in the last 7 days of life.^6^ Late hospice referral is associated with unmet needs, more concern about care, lower satisfaction, and greater levels of hospitalization and intensive care use in the last month of life.^7,8,9,10^ In addition, the rise in hospice use has been coupled with a rise in intensive care use at the end of life, indicating that some older adults may only be accessing hospice care after receiving intensive care.^11^

Prior research has sought to examine the pathway to care, or the site of care prior to hospice referral, to assess whether increased hospice use has changed overall patterns of care.^12^ The pathway to hospice may influence the timeliness of hospice care given that late hospice referrals often occur from the hospital setting.^13^ From 2011 to 2018, the site of care prior to hospice enrollment remained stable despite the overall increase in hospice use, although there was large variation by region.^12^ It is unclear what health system or insurance factors are driving this regional variation in the pathway to hospice.

One factor that may affect the pathway to hospice is Medicare Advantage (MA) enrollment. Given that MA is anticipated to insure the majority of Medicare beneficiaries in coming years, it is important to understand how MA enrollment is associated with hospice care.^14^ While hospice is currently carved out of MA, meaning that older adults in MA enrolling in hospice have their hospice benefits paid for as part of traditional Medicare (TM), MA plans may steer beneficiaries toward hospice care. Older adults in MA have historically enrolled in hospice at higher rates than those in TM, although the gap is narrowing.^1,2,15^ Nationally, the length of stay in hospice is either similar or longer for MA beneficiaries vs TM beneficiaries,^16^ although MA beneficiaries in the Veterans Affairs system had a shorter length of stay in hospice after adjusting for differences in clinical illness and sociodemographic characteristics.^17^ Hospice users have lower rates of hospitalization at the end of life, and studies have shown either lower or equivalent rates of hospitalization at the end of life between MA beneficiaries and TM beneficiaries depending on the population and the analytic approach.^15,17,18^

There are multiple challenges to comparing care patterns between decedents in MA and those in TM. Even among decedents, there are differences between those who were enrolled in MA and those in TM, with those in TM being sicker over the last year of life, with worse functional status and overall health and higher rates of dementia.^19^ Individuals are more likely to disenroll from MA in the last year of life, further complicating the comparison.^20^ Complete claims data on health care use in MA are not available, although an alternative approach can be used to examine inpatient, nursing facility, home health, and hospice care in both MA and TM.^16,21^ Medicare Advantage penetration varies widely by county, and the impact of MA may differ by regional penetration both within the MA population and in terms of spillover to the TM population.^22,23^ In addition, coverage across MA and TM is not homogenous, with some individuals in MA being enrolled in Special Needs Plans, such as those that serve dual-eligible beneficiaries, and some individuals in TM being enrolled in accountable care organizations (ACOs) that have incentives to reduce spending at the end of life.

We therefore aimed to compare the site of care prior to hospice enrollment among decedents using hospice in MA vs TM and to specifically assess whether decedents using hospice in MA were more likely to enroll in hospice from the community vs hospital or nursing home settings. As a secondary aim, we examined the association between MA vs TM enrollment and length of stay in hospice.

## Methods

This cross-sectional study followed the Strengthening the Reporting of Observational Studies in Epidemiology (STROBE) reporting guideline and was approved by the Brown University School of Public Heath institutional review board. Medicare claims data were used under a Centers for Medicare & Medicaid Services data use agreement, and the institutional review board of Brown University waived informed consent.

### Data and Cohort

This retrospective cross-sectional study used a 100% Medicare claims sample in calendar years 2011, 2013, 2016, and 2018 to identify decedents who first enrolled in hospice in the last 90 days of life. These years were selected to represent longitudinal trends. We excluded individuals with a hospice length of stay longer than 90 days because the study focus was on care patterns at the end of life. In our preliminary analysis, decedents in MA had an adjusted 0.7% lower proportion of hospice stays of more than 90 days compared with those in TM, so differences between the groups were negligible. For sensitivity analyses to account for differences in the decedent populations enrolled in MA vs TM, we separately examined only those with a hospice terminal diagnosis of cancer.

### Measures

The primary outcome variable, location of care prior to hospice enrollment, was derived from a modified Residential History File, an algorithm developed at Brown University that leverages a variety of claims and assessment data to capture the daily location of care for both those in MA and those in TM.^12,24,25^ This approach has been shown to correctly identify 94.5% of nursing home stays, 81.0% of hospitalizations, and 82.6% of Medicare beneficiaries at home compared with Part B place of service codes^24^ and correctly identify 90% of locations of death as identified using death certificates.^25^ Inpatient stays were identified using the Medicare Provider Analysis and Review (MedPAR) file, which contains data on 92% of MA hospitalizations.^26^ Nursing facility stays were identified using the Minimum Data Set, and home health was identified using the Outcome and Assessment Information Set, both of which are assessments conducted regardless of MA status. Given that hospice is carved out of MA, hospice care was captured among all decedents using fee-for-service hospice claims. The location of care prior to hospice enrollment was also derived from hospice claims, particularly because the MedPAR file does not contain information on all hospitalizations in MA. The location of care prior to hospice enrollment was categorized as hospital, nursing home, home with home health, and home without home health. Given that the hospice referral processes may take several days, individuals who enrolled in hospice and died within 3 days after hospital discharge to home health or 7 days after hospital discharge to a nursing home or home without home health were considered to be in the hospital prior to hospice enrollment. For regression models, the site of care prior to hospice enrollment was dichotomized as the community vs all other settings. We excluded individuals for whom we could not identify the site of care prior to hospice enrollment.

Enrollment in MA vs TM in the month of death was determined using the Medicare Beneficiary Summary File. Given the potential that decedents who were sicker may have disenrolled from MA within the last year of life, as a sensitivity analysis, MA enrollment was also determined by status 12 months prior to death. In addition, among those in TM, we assessed ACO enrollment over the year prior to death based on the final enrollment variable for 2016 and 2018 (the only years that these data were available).

Covariates for all analyses included decedent age at death, sex, race and ethnicity, year of death, dual eligibility with Medicaid, end-stage renal disease status, and hospice primary diagnosis (categorized as cancer, chronic obstructive pulmonary disease, congestive heart failure, dementia, cerebrovascular accident, and other). Race and ethnicity, including Hispanic; non-Hispanic Alaska Native or American Indian, Asian, Black, and White; and other, were based on self-report to the US Social Security Administration, classified by the Research Triangle Institute algorithm, and included in this study given evidence of differences in hospice use patterns by race and ethnicity.^27,28^ In addition, due to the potential impact of MA penetration, county-level MA penetration in 2018 was derived from publicly available files.

### Statistical Analysis

Data were analyzed from February 11, 2022, to October 24, 2022. We first assessed the distribution of site of care prior to hospice enrollment in MA vs TM over time and then assessed hospice length of stay by site of care prior to hospice enrollment. We then compared the sociodemographic characteristics and medical conditions of the population with community vs hospital site of care prior to hospice enrollment. To measure the association of MA enrollment with community vs other site of care prior to hospice, we used a logistic regression model with the aforementioned covariates and county-level fixed effects to account for the characteristics of the local MA market and the availability of hospice. We estimated this model for all counties and for counties stratified by the quintile of MA penetration and only for decedents in 2016 and 2018 (given that these were the years for which we had data on ACO enrollment).

We then conducted multiple sensitivity analyses to account for end-of-life disenrollment from MA and differences in the decedent populations in MA vs TM. First, we limited the cohort to individuals with cancer as the primary hospice diagnosis. Second, we defined MA and ACO status as the enrollment at 12 months prior to death. Third, we stratified by dual enrollment status with Medicaid.

We then assessed for differences in hospice length of stay between MA beneficiaries and TM beneficiaries using linear and logistic regression models to examine the mean length of stay (in days) and proportion of decedents with a hospice stay of 3 days or fewer in MA vs TM, respectively, accounting for all decedent characteristics and county-level fixed effects. Given the large sample size that could detect small statistically but not clinically meaningful differences, we emphasized effect sizes compared with statistically significant differences in our interpretation of the results. All analyses were conducted with Stata, version 17.0 (StataCorp LLC).

## Results

**Figure 1.**
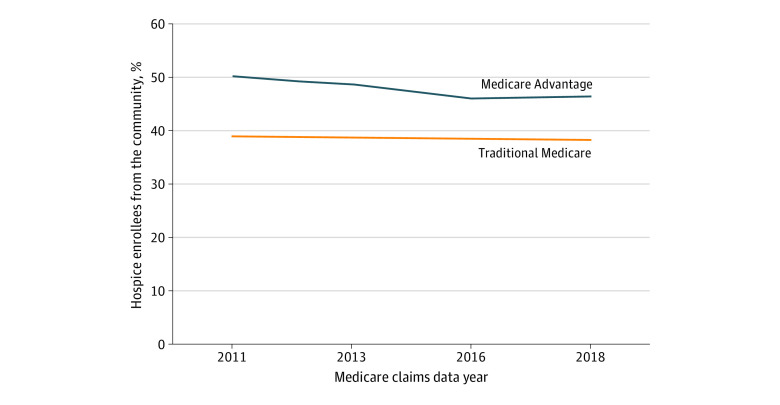
Proportion of Hospice Enrollees From the Community Over Time Medicare Advantage and traditional Medicare status was determined from insurance enrollment in the last month of life. Community is defined as the site of care prior to hospice enrollment being a noninstitutional setting with or without home health services compared with a hospital or nursing facility.

We identified 7 606 190 decedents in 2011, 2013, 2016, and 2018. After excluding decedents who did not have an identifiable site of care prior to hospice enrollment (4804 [0.6%]), did not enroll in hospice (3 773 595 [49.6%]), had a hospice stay longer than 90 days (661 448 [8.7%]), and resided in a county with fewer than 15 deaths (1384 [0.2%]), the final sample size was 3 164 959 (eFigure 4 in Supplement 1). The mean (SD) age was 83.1 (8.6) years, 55.8% were female, 44.2% were male, 4.9% were Hispanic, 0.3% were non-Hispanic Alaska Native or American Indian, 1.5% were non-Hispanic Asian, 7.5% were non-Hispanic Black, 85.0% were non-Hispanic White, 0.4% were other race and ethnicity, and 0.2% were unknown. A total of 28.8% decedents were enrolled in MA (Table 1). Characteristics of decedents by insurance type are shown in eTable 4 in Supplement 1. The proportion of those with missing data on the site of care prior to hospice enrollment did not vary by insurance type (eTable 7 in Supplement 1). Of those in TM, 48.9% enrolled in hospice compared with 52.8% in MA. From 2011 to 2018, the proportion of hospice beneficiaries enrolling from the community was higher in MA, although the gap between MA and TM narrowed over time from an unadjusted 11.1% higher rate of community enrollment in MA vs TM in 2011 (50.1% vs 39.0%) to 8.1% in 2018 (46.4% vs 38.3%) (Figure 1). There was minimal difference between decedents in TM not in an ACO vs those in an ACO at the end of life (eFigure 1 in Supplement 1). As shown in Table 1, decedents who enrolled in hospice from the community vs other settings were more likely to be male (46.4% vs 42.7%), less likely to have dual eligibility for Medicaid (11.5% vs 27.0%), less likely to be non-Hispanic Black (6.5% vs 8.3%), and more likely to have cancer as their primary hospice diagnosis (40.1% vs 24.1%). Mean (SD) length of stay in hospice was shortest for decedents enrolling from acute care hospitals (5.9 [7.1] days in TM; 6.0 [7.1] days in MA) and longest for those enrolling in hospice from the community without home health (10.7 [10.1] days in TM; 10.2 [9.5] days in MA), with smaller differences by MA vs TM status compared with site of care prior to enrollment (Table 2). A similar hospice length of stay by site of care persisted even when the sample was stratified by insurance type (eTable 5 in Supplement 1), when length of stay was examined only for those with cancer (eTable 6 in Supplement 1), and when the distribution across length-of-stay categories was examined (eFigure 3 in Supplement 1).

**Table 1.** Characteristics of Decedents From the Community vs All Other Sites of Care Prior to Hospice Enrollment^a^

Characteristic	Decedents^b^		
	Overall (N = 3 164 959)	From the community (n = 1 307 590)	From all other sites (n = 1 857 369)
Age, mean (SD)	83.1 (8.6)	82.8 (8.7)	83.4 (8.5)
Sex			
Female	1 764 471 (55.8)	700 956 (53.6)	1 063 515 (57.3)
Male	1 400 488 (44.2)	606 634 (46.4)	793 854 (42.7)
Medicaid dual eligibility	652 391 (20.6)	150 961 (11.5)	501 430 (27.0)
Race and ethnicity			
Hispanic	155 310 (4.9)	64 091 (4.9)	91 219 (4.9)
Non-Hispanic			
Alaska Native or American Indian	10 286 (0.3)	4217 (0.3)	6069 (0.3)
Asian	48 675 (1.5)	20 901 (1.6)	27 774 (1.5)
Black	238 745 (7.5)	84 938 (6.5)	153 807 (8.3)
White	2 690 332 (85.0)	1 123 747 (85.9)	1 566 585 (84.3)
Other^c^	13 844 (0.4)	6111 (0.5)	7733 (0.4)
Unknown	7767 (0.2)	3585 (0.3)	4182 (0.2)
Hospice diagnosis			
ESRD	72 480 (2.3)	23 211 (1.8)	49 269 (2.7)
Cancer	972 934 (30.7)	524 874 (40.1)	448 060 (24.1)
Dementia	791 016 (25.0)	306 200 (23.4)	484 816 (26.1)
COPD	193 239 (6.1)	77 712 (5.9)	115 527 (6.2)
CHF	332 023 (10.5)	128 463 (9.8)	203 560 (11.0)
CVA	123 093 (3.9)	26 331 (2.0)	96 762 (5.2)
All other diagnoses	885 411 (28.0)	295 872 (22.6)	589 539 (31.7)
County-level MA penetration, mean (SD)	31.2 (14.4)	31.7 (14.5)	30.9 (14.3)

Abbreviations: CHF, congestive heart failure; COPD, chronic obstructive pulmonary disease; CVA, cerebrovascular accident; ESRD, end-stage renal disease; MA, Medicare Advantage.

^a^ Data are from Medicare claims data from 2011, 2013, 2016, and 2018.

^b^ Data are reported as number (percentage) of decedents unless otherwise indicated.

^c^ “Other” was self-reported by participants.

**Table 2.** Hospice Length of Stay by Site of Care Prior to Hospice^a^

Site of care	Hospice enrollees, No.	Hospital length of stay, d			
		Traditional Medicare		Medicare Advantage	
		Mean (SD)	Median (IQR)	Mean (SD)	Median (IQR)
Acute care hospital	1 211 343	5.9 (7.1)	3 (1-8)	6.0 (7.1)	4 (1-8)
Other hospital	26 735	6.8 (7.9)	4 (2-9)	6.2 (7.5)	4 (1-8)
Skilled nursing facility	124 361	8.4 (8.7)	6 (2-12)	9.5 (9.9)	7 (3-13)
Nursing home	495 745	9.1 (9.1)	6 (2-13)	8.9 (9.0)	6 (2-13)
Community with home health	234 735	10.0 (9.3)	7 (3-15)	9.4 (8.8)	7 (3-13)
Community without home health	1 073 424	10.7 (10.1)	8 (3-16)	10.2 (9.5)	8 (3-15)
Overall	3 166 343	8.3 (9.0)	5 (2-12)	8.4 (8.8)	6 (2-12)

^a^ Data are from Medicare claims data from 2011, 2013, 2016, and 2018. Traditional Medicare and Medicare Advantage status were determined at the last month of life.

**Figure 2.**
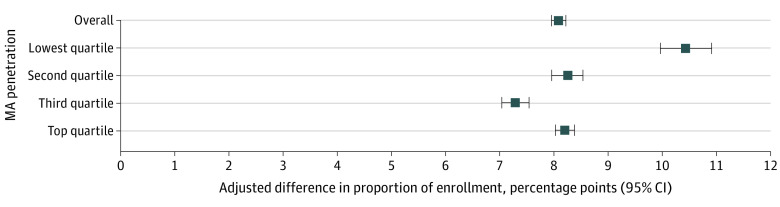
Adjusted Difference in the Proportion of Community Hospice Enrollment in Medicare Advantage (MA) vs Traditional Medicare Overall and by County-Level Quartile of MA Penetration Data are from Medicare claims data from 2011, 2013, 2016, and 2018. The model was adjusted for age at death, sex, race and ethnicity, year of death, Medicaid status, end-stage renal disease status, and hospice primary diagnosis (categorized as cancer, chronic obstructive pulmonary disease, congestive heart failure, dementia, cerebrovascular accident, and all other diagnoses). Whiskers indicate 95% CIs.

In the primary model estimating community hospice enrollment (Figure 2), after adjusting for covariates and county-level fixed effects, decedents in MA vs those in TM had an estimated 8.09–percentage point (95% CI, 7.96-8.21 percentage points) higher rate of hospice enrollment from the community vs all other sites, with a U-shaped pattern when stratified by county-level MA penetration. Counties with the lowest MA penetration rates had the largest difference (10.44 percentage points; 95% CI, 9.97-10.91 percentage points) in the proportion of community hospice enrollment (Figure 2). In multiple sensitivity analyses, results were largely similar, including minimal differences between those in TM enrolled in ACOs vs those not enrolled in ACOs (eTable 3 in Supplement 1).

In the full cohort, MA enrollment was associated with a 0.29-day (95% CI, 0.24-0.34 days) longer hospice stay compared with enrollment in TM, with no differences between enrollment in ACOs and TM (Table 3). Among decedents who switched between MA and TM in the last year of life (eFigure 2 in Supplement 1), those who remained in MA the entire last year of life had an estimated 8.1–percentage point (95% CI, 7.9-8.2 percentage points) higher proportion of community hospice enrollment vs those who stayed in TM the entire year. Decedents who joined MA in the last year of life had an estimated 5.2–percentage point (95% CI, 4.9-5.6 percentage points) higher proportion of community hospice enrollment vs those who stayed in MA the entire year. However, decedents who left MA had an estimated 3.7–percentage point (95% CI, 4.1-3.3 percentage points) lower proportion of community hospice enrollment vs those who stayed in MA the entire year.

**Table 3.** Adjusted Differences in Hospice Length of Stay and Proportion of Short Hospice Stays by Decedent Insurance Type^a^

Measure	Traditional Medicare^b^	Medicare Advantage	Accountable care organization
Length of stay, mean (95% CI), d	1 [Reference]	0.03 (0.01 to 0.05)	−0.20 (−0.24 to −0.16)
Proportion with short stay, % (95% CI)	1 [Reference]	−0.61 (−0.74 to −0.48)	0.67 (0.46 to 0.88)

^a^ Data are from Medicare claims data from 2011, 2013, 2016, and 2018. A short hospice stay was defined as 3 or fewer days. Analyses were adjusted for year of death, end-stage renal disease, age, sex, race and ethnicity, Medicaid enrollment, and county-level fixed effects.

^b^ Not in an accountable care organization.

## Discussion

In this national cross-sectional study of Medicare decedents, we found that hospice enrollees in MA had substantially different hospice use patterns, with greater enrollment from community settings, compared with their counterparts in TM. While we found that hospice length of stay was longer for those enrolling from the community vs hospital or nursing home settings, we overall found that hospice length of stay among MA enrollees was only slightly longer than for those in TM. Given the substantial increase in the number of MA beneficiaries, including those at the end of life, this finding fills a gap in our understanding of how MA is associated with care and yet leaves many unanswered questions, particularly as to why there was little change in hospice length of stay by insurance type despite the increase in persons enrolling from the community.

It is unclear why MA beneficiaries were more likely to enroll in hospice from the community. Medicare Advantage plans are incentivized to control costs of care. Given that spending on hospice is carved out of MA plans, MA has an even greater incentive to proactively identify older adults likely to be hospice eligible and steer them toward hospice care before a change in their medical condition that may trigger hospitalizations. Prior qualitative research has shown that MA plans use active case management for postacute care utilization^29^; thus, it is possible that similar approaches for MA beneficiaries who have received postacute care in prior months may be associated with increases in their enrollment in hospice. Alternatively, it is possible that physicians or care systems in MA networks are more incentivized to enroll eligible patients in hospice, particularly if this results in avoided inpatient admissions. Future mixed-methods work is needed to understand the mechanisms that MA plans use to shape hospice use. This study’s findings may inform efforts to increase hospice enrollment among all eligible beneficiaries to ensure that there are no adverse consequences of particular populations being the focus of hospice enrollment, such as live discharges of patients from hospice or increased pressure about decision-making for patients and families.^30^

Our finding of increased hospice length of stay for those with hospice enrollment from the community aligns with research that has demonstrated that hospice enrollment that follows hospital stays is often short.^31^ However, it is not clear why there were no major differences in length of stay between MA and TM among persons enrolled in hospice. We attempted a series of analyses to examine the lack of difference, including examining the association between insurance and length of stay among only those with cancer to account for potentially different patient populations and stratifying by insurance type to examine the association between site of care prior to hospice enrollment and length of stay in case MA influenced hospice enrollment differently across settings. Even when examining only short stays in hospice, we found no difference, in contrast with prior research^17^ that found that when adjusting for beneficiary characteristics, MA enrollees had a higher rate of short (≤3-day) hospice admissions.

That length of stay in hospice did not change despite the different enrollment setting in MA deserves future, focused research. It is possible that differences are explained by different populations selectively enrolling in or disenrolling from MA plans, despite our attempts to adjust for differences in case mix. It is possible that MA also operates differently in terms of steering beneficiaries to hospice settings that are more likely to enroll people in the community but also enroll people at a later time after the hospice referral. We found that the difference in length of stay between decedents admitted from the community vs an acute care hospital was smaller among MA decedents than among TM decedents (Table 2). While these differences were not large enough to fully explain why there were only small differences in overall length of hospice stay between TM and MA, they warrant an in-depth examination of how MA functions differently across settings to steer beneficiaries to hospice. Our findings suggest that challenges exist regarding increasing hospice length of stay under the current benefit design given the persistently short hospice length of stay even when the setting of enrollment varied.

The proportion of community hospice enrollment among individuals in MA was not larger in the quartile of counties with the highest level of MA penetration, as we hypothesized there would be. This may be because of other factors associated with low MA penetration, such as rurality or decedent demographics (eTables 1 and 2 in Supplement 1), spillover from MA to TM in regions with high MA penetration, or variation in MA plans across regions. Further research should examine how different MA plan types and structures influence hospice use. Finally, our results also showed that beneficiaries who left MA plans had the lowest rates of community hospice enrollment. Given recent data highlighting the high rates of disenrollment from MA at the end of life,^20^ further monitoring and research should assess why individuals leave MA at the end of life and potentially hold MA plans accountable for the quality of care of this population if they are disenrolling because MA plans are not meeting their needs.

### Limitations

This study has limitations. While we examined site of care prior to hospice and length of stay in hospice, we did not examine variation in hospice care quality or delivery of palliative care services prior to hospice enrollment, which may be associated with care quality. We examined the place of care prior to hospice enrollment, but this may not have captured the full trajectory of care for those who had multiple transitions in care settings in the last year of life. While we used an innovative approach to assess care patterns in MA prior to hospice enrollment and used the place of care derived from hospice claims as well as other sources, we may have underreported some hospital stays in MA as the MedPAR file does not contain all stays for MA enrollees. We also did not differentiate between those in the community who were in assisted living or other domiciliary settings and those who were in private homes. As we used claims-based measures of health care use, we did not capture the perceptions of older adults at the end of life or bereaved family and friends. Further studies relying on direct patient or bereaved proxy reports of quality are necessary to assess how MA impacts the quality of care overall and particularly through changing the pathway to hospice care. Furthermore, while we used multiple approaches to account for differences in the populations enrolled in MA vs TM, there may still be differences in the clinical trajectories of these groups at the end of life, and these differences may influence who selects into or disenrolls from MA. There may also be differences across regions, such as patterns shaped by hospice availability, and while our study design that included county-level fixed effects reduced bias from regional differences, it did not allow us to examine them. Finally, while we measured the associations between MA enrollment and the site of care prior to hospice, further research is needed to assess causality.

## Conclusions

In this cross-sectional study, compared with TM beneficiaries, MA beneficiaries were more likely to enroll in hospice from community settings vs following inpatient stays. However, hospice length of stay was not substantially different between MA and TM. While differences in hospice use between MA beneficiaries and TM beneficiaries enrolled in ACOs were minimal, MA plans in all analyses had a significant association with hospice enrollment from a community setting. Given the growth in the MA program, better understanding of how MA plans influence the quality of end-of-life care among decedents is needed.

## Notes

Supplement 1. **eTable 1.** Characteristics of Decedents Across County-Level Quartiles of MA Penetration **eFigure 1.** Proportion of Hospice Enrollees From the Community (With and Without Home Health) Over Time, Including Those in TM in ACOs (“ACO”) vs TM Not in ACOs (“TM”) **eTable 2.** Adjusted Increase in the Proportion of Community vs Hospital Enrollment, Results for All Sensitivity Analyses **eFigure 2.** Adjusted Difference in the Proportion of Community Hospice Enrollment in Each Subgroup vs Those Remaining in TM the Entire Last Year of Life (N = 2 194 624) **eTable 3.** Adjusted Difference in the Proportion of Community Hospice Enrollment in MA vs TM Non-ACO vs TM in ACOs, 2016 and 2018 **eTable 4.** Characteristics of Decedents by Insurance Status **eTable 5.** Adjusted Model Predicting Hospice LOS, Stratifying by Insurance Type **eTable 6.** Adjusted Length of Stay in Hospice by Insurance Type, Only Among Those With a Cancer Diagnosis **eFigure 3.** Distribution of Hospice Length of Stay Across Insurance Types **eFigure 4.** Flowchart of Sample Size and Exclusion Criteria for Study **eTable 7.** Number and Proportion Missing Data on Hospice Pathway, by Insurance Type

Supplement 2.
Data Sharing Statement
