# Supplementary material for: Comparison of the Pathway to Hospice Enrollment Between Medicare Advantage and Traditional Medicare
Source: JAMA Health Forum. 2023 Feb 17;4(2):e225457. doi: 10.1001/jamahealthforum.2022.5457 (PMC9938424; doi:10.1001/jamahealthforum.2022.5457)
Supplement: Supplement 1. — eTable 1. Characteristics of Decedents Across County-Level Quartiles of MA Penetration eFigure 1. Proportion of Hospice Enrollees From the Community (With and Without Home Health) Over Time, Including Those in TM in ACOs (“ACO”) vs TM Not in ACOs (“TM”) eTable 2. Adjusted Increase in the Proportion of Community vs Hospital Enrollment, Results for All Sensitivity Analyses eFigure 2. Adjusted Difference in the Proportion of Community Hospice Enrollment in Each Subgroup vs Those Remaining in TM the Entire Last Year of Life (N = 2 194 624) eTable 3. Adjusted Difference in the Proportion of Community Hospice Enrollment in MA vs TM Non-ACO vs TM in ACOs, 2016 and 2018 eTable 4. Characteristics of Decedents by Insurance Status eTable 5. Adjusted Model Predicting Hospice LOS, Stratifying by Insurance Type eTable 6. Adjusted Length of Stay in Hospice by Insurance Type, Only Among Those With a Cancer Diagnosis eFigure 3. Distribution of Hospice Length of Stay Across Insurance Types eFigure 4. Flowchart of Sample Size and Exclusion Criteria for Study eTable 7. Number and Proportion Missing Data on Hospice Pathway, by Insurance Type [file jamahealthforum-e225457-s001.pdf]

Supplemental Online Content

Ankuda CK, Belanger E, Bunker J, et al. Comparison of the pathway to hospice enrollment between Medicare Advantage and traditional Medicare. *JAMA Health Forum*. Published online 2023;4(2):e225457. doi:10.1001/jamahealthforum.2022.5457

eTable 1. Characteristics of Decedents Across County-Level Quartiles of MA Penetration

eFigure 1. Proportion of Hospice Enrollees From the Community (With and Without Home Health) Over Time, Including Those in TM in ACOs ("ACO") vs TM Not in ACOs ("TM")

eTable 2. Adjusted Increase in the Proportion of Community vs Hospital Enrollment, Results for All Sensitivity Analyses

eFigure 2. Adjusted Difference in the Proportion of Community Hospice Enrollment in Each Subgroup vs Those Remaining in TM the Entire Last Year of Life (N = 2 194 624)

eTable 3. Adjusted Difference in the Proportion of Community Hospice Enrollment in MA vs TM Non-ACO vs. TM in ACOs, 2016 and 2018

eTable 4. Characteristics of Decedents by Insurance Status

eTable 5. Adjusted Model Predicting Hospice LOS, Stratifying by Insurance Type

eTable 6. Adjusted Length of Stay in Hospice by Insurance Type, Only Among Those With a Cancer Diagnosis

eFigure 3. Distribution of Hospice Length of Stay Across Insurance Types

eFigure 4. Flow Chart of Sample Size and Exclusion Criteria for Study

eTable 7. Number and Proportion Missing Data on Hospice Pathway, by Insurance Type

This supplemental material has been provided by the authors to give readers additional information about their work.

**eTable 1**. Characteristics of Decedents Across County-Level Quartiles of MA Penetration				
Variable	1st Quartile N=482,116	2nd Quartile N=691,127	3rd Quartile N=717,474	4th Quartile N=1,243,086
MA rate, mean (IQR)	10.0 (7.3, 10.5, 12.8)	20.7 (18.1, 20.7, 23.2)	30.6 (25.8, 30.4, 33.1)	45.9 (40.1, 44.1, 49.9)
Age, mean (IQR)	83.0 (77, 84, 90)	82.9 (76, 84, 89)	83.0 (76, 84, 90)	83.8 (77, 84, 90)
Male, mean (IQR)	43.9 (43.8, 44.1)	44.0 (43.9, 44.2)	44.4 (44.3, 44.5)	44.4 (44.3, 44.5)
Dual status, %	23.0 (22.9, 23.2)	23.4 (23.3, 23.5)	20.9 (20.8, 21.0)	17.9 (17.9, 18.0)
White, %	88.3 (88.2, 88.4)	87.8 (87.7, 87.8)	87.3 (87.3, 87.4)	80.8 (80.8, 80.9)
Black, %	7.3 (7.2, 7.3)	8.0 (7.9, 8.0)	7.3 (7.2, 7.4)	7.5 (7.4, 7.5)
Hispanic, %	2.4 (2.3, 2.4)	2.7 (2.7, 2.8)	3.6 (3.6, 3.7)	7.9 (7.9, 8.0)
Asian, %	2.0 (2.0, 2.1)	1.5 (1.5, 1.6)	1.8 (1.7, 1.8)	3.8 (3.7, 3.8)
ESRD, %	2.2 (2.1, 2.2)	2.3 (2.2, 2.3)	2.3 (2.2, 2.3)	2.4 (2.3, 2.4)
Cancer, %	31.8 (31.7, 32.0)	31.4 (31.3, 31.5)	30.1 (30.0, 30.2)	30.3 (30.2, 30.4)
Dementia, %	22.1 (22.0, 22.2)	24.4 (24.3, 24,5)	25.5 (25.4, 25.6)	26.2 (26.1, 26.3)
COPD, %	6.2 (6.1, 6.3)	6.4 (6.3, 6.4)	6.3 (6.2, 6.3)	5.8 (5.8, 5.9)
CHF, %	9.9 (9.8, 10.0)	10.4 (10.3, 10.4)	10.8 (10.7, 10.8)	10.7 (10.6, 10.7)
CVA, %	4.1 (4.1, 4.2)	3.8 (3.8, 3.9)	3.8 (3.8, 3.9)	3.9 (3.8, 3.9)
All other diagnoses, %	29.1 (28.9, 29.2)	27.7 (27.5, 27.8)	28.0 (27.9, 28.1)	27.7 (27.6, 27.7)
Died in 2011, %	39.1 (38.9, 39.2)	26.2 (26.1, 26.3)	18.0 (17.9, 18.1)	15.5 (15.4, 15.6)
Died in 2013, %	29.3 (29.2, 29.4)	26.0 (25.9, 25.1)	24.6 (24.5, 24.7)	20.4 (20.3, 20.4)
Died in 2016, %	18.8 (18.6, 18.9)	26.6 (26.5, 26.7)	27.0 (26.9, 27.0)	27.9 (27.8, 27.9)
Died in 2018, %	12.9 (12.8, 13.0)	21.2 (21.1, 21.2)	30.4 (30.3, 30.5)	36.3 (36.2, 36.4)

© 2023 Ankuda CK et al. *JAMA Health Forum*.

eFigure 1. Proportion of Hospice Enrollees From the Community (With and Without Home Health) Over Time, Including Those in TM in ACOs (“ACO”) vs TM Not in ACOs (“TM”)

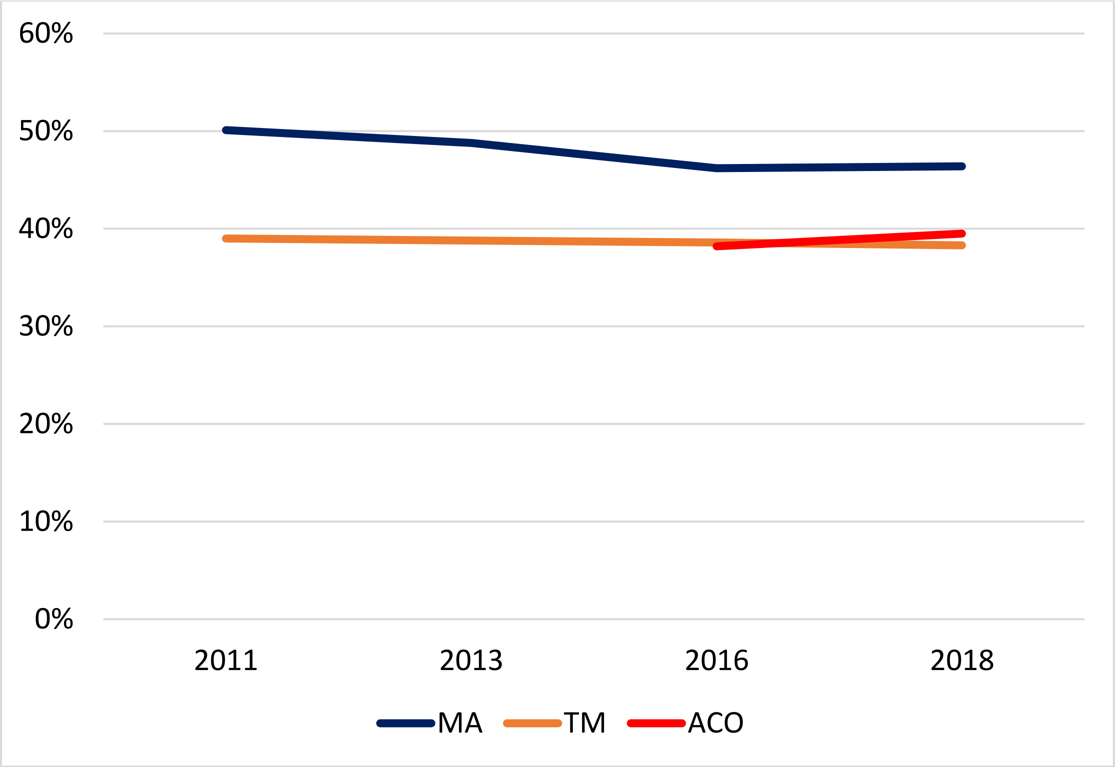

Legend: Data source: Medicare claims data from 2011, 2013, 2016 and 2018. MA= Medicare Advantage, TM= traditional Medicare. MA/TM status determined from insurance enrollment in the last month of life. Community is defined as site of care prior to hospice enrollment being a non-institutional setting with or without home health services, compared to a hospital or nursing facility.

© 2023 Ankuda CK et al. *JAMA Health Forum*.

**eTable 2.** Adjusted Increase in the Proportion of Community vs Hospital Enrollment, Results for All Sensitivity Analyses			
	TM- not ACO	MA	TM- ACO
Primary model	Reference	8.09% (7.96%-8.21%)	0.80% (0.60%-1.01%)
Cohort with cancer as hospice primary diagnosis	Reference	6.64% (6.41%-6.87%)	0.55% (0.17%-0.93%)
MA/ACO status determined 12 months prior to death	Reference	7.22% (7.10%-7.34%)	0.53% (0.33%-0.74%)
Non-duals	Reference	7.82% (7.68%-7.96%)	0.13% (-0.11%-0.36%)
Duals	Reference	8.52% (8.29%-8.75%)	3.58% (3.17%-4.00%)
TM= Traditional Medicare, ACO=Accountable Care Organization, MA= Medicare Advantage. For all models, covariates included decedent age at death, sex, race/ethnicity, year of death, dual status, end-stage renal disease status, and hospice primary diagnosis (categorized as cancer, chronic obstructive lung disease (COPD), congestive heart failure (CHF), dementia, cerebral vascular injury (CVA), and other).			

© 2023 Ankuda CK et al. *JAMA Health Forum*.

eFigure 2. Adjusted Difference in the Proportion of Community Hospice Enrollment in Each Subgroup vs Those Remaining in TM the Entire Last Year of Life (N = 2 194 624)

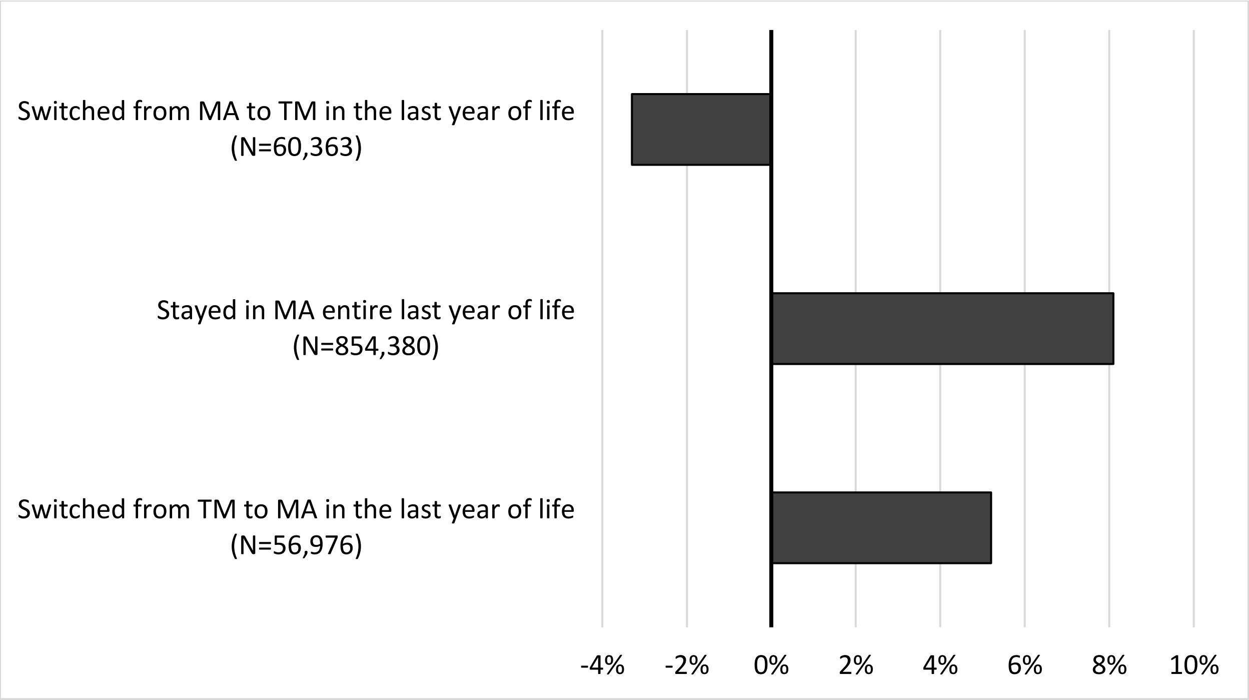

Legend: Data source: Medicare claims data from 2011, 2013, 2016 and 2018. Proportions of >0% indicate greater hospice enrollment in the indicated population vs. those remaining in TM the entire year. Proportions of <0% indicate lower hospice enrollment in the indicated population vs. those remaining in TM the entire year. Model adjusted for age at death, sex, race/ethnicity, year of death, Medicaid status, end-stage renal disease status, and hospice primary diagnosis (categorized as cancer, chronic obstructive lung disease, congestive heart failure, dementia, cerebral vascular injury, or other) and county-level fixed effects.

© 2023 Ankuda CK et al. *JAMA Health Forum*.

**eTable 3.** Adjusted Difference in the Proportion of Community Hospice Enrollment in MA vs TM Non-ACO vs. TM in ACOs, 2016 and 2018		
	OR (95% CI)	p-value
TM non-ACO	Reference	
MA	7.5% (7.3%-7.6%)	<0.001
TM in ACO	0.5% (0.3%-0.8%)	<0.001
TM= Traditional Medicare, MA=Medicare Advantage, ACO= Accountable Care Organization		

© 2023 Ankuda CK et al. *JAMA Health Forum*.

eTable 4. Characteristics of Decedents by Insurance Status

Characteristic, N (%):	MA<br>N=911,182	TM, ACO<br>N=259,793	TM, not ACO<br>N=1,993,984
Age, mean	82.6	83.4	83.3
Male	45.5%	44.2%	43.7%
Dual status	18.9%	17.1%	21.9%
Race and ethnicity:			
Non-Hispanic White	80.7%	88.7%	86.5%
Non-Hispanic Black	9.0%	6.0%	7.1%
Hispanic	7.5%	3.1%	4.0%
Non-Hispanic Asian	2.9%	2.2%	2.4%
Hospice diagnosis:			
ESRD	1.9%	2.3%	2.5%
Cancer	32.5%	32.2%	29.7%
Dementia	24.0%	27.1%	25.2%
COPD	6.2%	6.0%	6.1%
CHF	10.5%	13.3%	10.1%
CVA	3.9%	3.3%	4.0%
All other diagnoses	27.2%	24.4%	28.8%

Data source: Medicare claims data from 2011, 2013, 2016 and 2018. IQR=interquartile range, ESRD= end-stage renal disease, COPD= chronic obstructive pulmonary disease, CHF= congestive heart failure, CVA= cerebrovascular accident.

© 2023 Ankuda CK et al. *JAMA Health Forum*.

eTable 5. Adjusted Model Predicting Hospice LOS, Stratifying by Insurance Type

A. Among all insurance types		
Site of care prior to hospice:	Coefficient	95% Confidence Interval
Acute care hospital	(Reference)	(Reference)
Other hospital	0.81	0.70-0.91
Skilled nursing facility	2.31	2.26-2.36
Nursing home	2.85	2.82-2.89
Community with home health	3.79	3.75-3.83
Community without home health	4.42	4.40-4.45
**B. Among those in MA**		
Site of care prior to hospice:	Coefficient	95% Confidence Interval
Acute care hospital	(Reference)	(Reference)
Other hospital	0.45	0.16-0.75
Skilled nursing facility	3.28	2.87-3.68
Nursing home	2.59	2.53-2.64
Community with home health	3.17	3.09-3.26
Community without home health	3.93	3.89-3.97
**A. Among those in TM**		
Site of care prior to hospice:	Coefficient	95% Confidence Interval
Acute care hospital	(Reference)	(Reference)
Other hospital	0.88	0.76-1.00
Skilled nursing facility	2.40	2.34-2.45
Nursing home	2.97	2.93-3.01
Community with home health	3.98	3.93-4.03
Community without home health	4.65	4.62-4.68

Data source: Medicare claims data from 2011, 2013, 2016 and 2018. Model is a linear regression predicting length of stay with county-level fixed effects, adjusting for year, insurance type (in Model A), age, sex, race, hospice diagnosis, dual status

© 2023 Ankuda CK et al. *JAMA Health Forum*.

**eTable 6.** Adjusted Length of Stay in Hospice by Insurance Type, Only Among Those With a Cancer Diagnosis		
	coefficient (95% CI)	p-value
TM non-ACO	Reference	
MA	-0.05 (-0.09- -0.01)	0.02
TM in ACO	-0.25 (-0.32- -0.19)	<0.001
TM= Traditional Medicare, MA=Medicare Advantage, ACO= Accountable Care Organization. Adjusting for year, age, sex, race, dual status, county-level fixed effects.		

© 2023 Ankuda CK et al. *JAMA Health Forum*.

eFigure 3. Distribution of Hospice Length of Stay Across Insurance Types

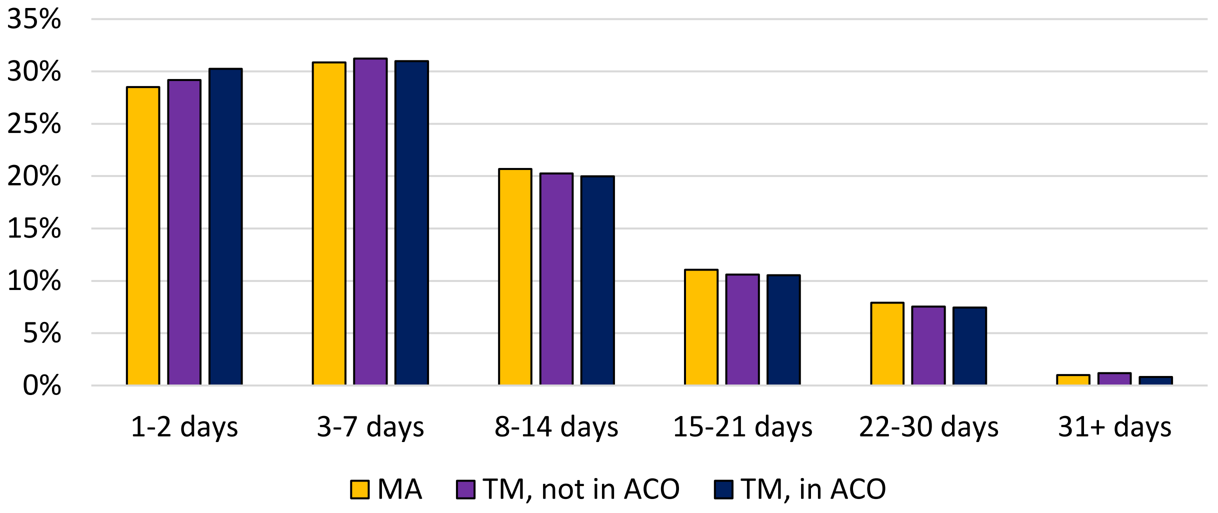

© 2023 Ankuda CK et al. *JAMA Health Forum*.

eFigure 4. Flow Chart of Sample Size and Exclusion Criteria for Study

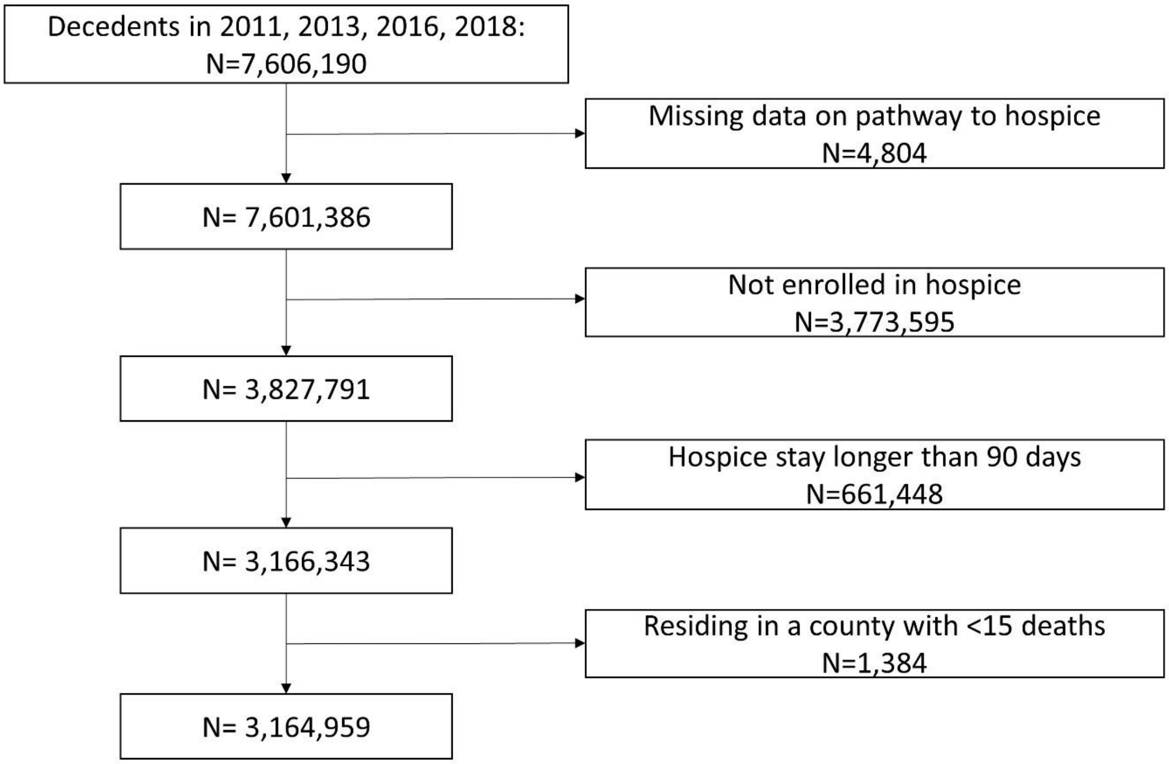

© 2023 Ankuda CK et al. *JAMA Health Forum*.

**eTable 7.** Number and Proportion Missing Data on Hospice Pathway, by Insurance Type		
	N	%
TM, not in ACO	3,236/4,974,247	0.07%
MA	1,234/2,081,186	0.06%
TM, in ACO	334/550,757	0.06%

© 2023 Ankuda CK et al. *JAMA Health Forum*.
