# Supplementary material for: Comparison of the Pathway to Hospice Enrollment Between Medicare Advantage and Traditional Medicare
Source: JAMA Health Forum. 2023 Feb 17;4(2):e225457. doi: 10.1001/jamahealthforum.2022.5457 (PMC9938424; doi:10.1001/jamahealthforum.2022.5457)
Supplement: Supplement 2. — Data Sharing Statement [file jamahealthforum-e225457-s002.pdf]

Data Sharing Statement

Ankuda. Comparison of the Pathway to Hospice Enrollment Between Medicare Advantage and Traditional Medicare. *JAMA Health Forum*. Published February 17, 2023. doi:10.1001/jamahealthforum.2022.5457

Data
Data available: No

Additional Information
Explanation for why data not available: The data is Medicare claims data and therefore protected under a data use agreement from sharing. Analytic code is available at https://doi.org/10.26300/3agx-c091.
